# Equity and unmet need of non-communicable diseases services in Saudi Arabia using a National Household Survey (2019)

**DOI:** 10.1186/s12913-024-10787-6

**Published:** 2024-03-16

**Authors:** Maha Alattas, Sarah Gordon, Lora L. Sabin, Fadi El-jardali, Veronika J. Wirtz

**Affiliations:** 1 Department of Global Health, Boston University School of Public Health, Boston, MA USA; 2 Department of Community Medicine, King Abdulaziz University, Jeddah, Saudi Arabia; 3 Department of Health Law, Policy and Management, Boston University School of Public Health, Boston, MA USA; 4 American University of Beirut, Beirut, Lebanon

**Keywords:** Health equity, Objective unmet need, Non-communicable disease, Primary care, Services utilization, Saudi Arabia

## Abstract

**Background:**

Saudi Arabia is implementing a comprehensive health system transformation in health services provision, governance, and financing. Given the high burden of non-communicable diseases (NCD), a key objective of the transformation is to integrate NCD prevention and treatment into primary care. The study objectives were to assess primary care service use for treatment of NCDs, to quantify existing inequities in preventive services utilization, and to identify regional and sociodemographic factors associated with these inequities.

**Methods:**

Using the 2019 Kingdom of Saudi Arabia World Health Survey, multivariable logistic regression models were conducted to identify predictors of utilization of primary care services for NCD prevention and treatment, unmet need among those with a diagnosis of diabetes, hypertension, or dyslipidemia, and unmet need in breast and cervical cancer screening.

**Results:**

Among those with an NCD diagnosis, living in a high-income household was associated with a lower probability of having an unmet need compared to those in low-income households. Furthermore, rural residents were less likely to have an unmet need compared to urban residents (OR 0.58, *p*=0.029). Individuals without a perceived need for healthcare within the last 12 months had three times the probability of unmet need in comparison to those with such a perceived need (*p*<0.001). Women in all regions had a lower probability of ever having a mammogram compared to women in the central regions around Riyadh. Women with an education above a secondary level had five times the odds of undergoing cervical cancer screening and three times the likelihood of ever having a mammogram (*P*=0.012, *p*=0.02) than other women. Compared to women in low-income households, those in middle (OR 1.99, *P*=0.026), upper middle (OR 3.47, *p*<0.001), or high-income households (OR 2.59, *p*<0.001) had a higher probability of having had cervical cancer screening.

**Conclusions:**

Inequities in NCD treatment and prevention services’ utilization in Saudi Arabia are strongly associated with region of living, population density, wealth, income, education and perceived need for health care. More research is needed to better understand the extent of unmet primary care needs for NCD and how to address the underlying contributing factors to access inequities.

**Supplementary Information:**

The online version contains supplementary material available at 10.1186/s12913-024-10787-6.

## Background

Non-communicable diseases (NCDs) represent a major disease burden globally, and account for 73.2% of all deaths in Saudi Arabia [1]. One-quarter of disability-adjusted life years (DALYs) in Saudi Arabia are attributable to cardiovascular diseases (CVDs), diabetes, chronic respiratory diseases, and cancers [2, 3]. CVDs contribute 37% of NCD-related mortality, which is the largest single contributor to mortality [2, 3]. The disease burden related to metabolic risk factors, including high fasting plasma glucose, high body mass index, and high low density lipoprotein (LDL), has been increasing over the past decade [4]. This burden of NCDs is expected to continue to grow rapidly in coming years [3].

To address the increasing NCD burden, rising costs of health care, and demographic shifts that include both longer life expectancy and a growing youth population, the Saudi government launched a major health system transformation in 2017 with the goal of improving health outcomes and reducing health care costs. Key elements of the health system transformation include decentralizing health care service provision through the implementation of “Health Clusters.” The clusters will operate as accountable care organizations by shifting regulatory authority to the regional level to better address population health needs in each defined catchment area. In addition, the mechanism of health care financing is supposed to shift from a centralized general line-item budget to a capitated budget. Health financing will also move from the Ministry of Health to the National Health Insurance Institute, which will be the leading payer for public services [5].

Alongside this transformation is an ongoing reform initiative that focuses on better integrating NCD prevention and treatment services into primary care [6]. Historically, the referral pathways had weak linkages between primary and secondary and tertiary care. While aspects of this weakness are being resolved with the recent establishment of the Ministry of Health Medical Referral Program [7, 8], the intent of the transformation is to further enhance the continuity of care across different levels of the health care system. Strengthening primary care infrastructure may also expand access to preventive and treatment services for NCDs, such as breast cancer screening, leading to improved health outcomes for people with NCDs.

While the term “fairness” is part of the health Law of Governance in the Kingdom, there is no single standard term for equity in Arabic. Health is a right given by the government in Saudi Arabia. However, there is no unified Health equity framework that has been used traditionally or highlighted in health policy documents in Saudi [9]. Recently, the Saudi Health Council published a national framework for health status and health system performance indicators, intending it to serve as roadmap for improving the health system. While it includes equity as a dimension, no definition of “equity” is included [10].

Published studies in the literature have focused mainly on assessing the disparities and inequities of the prevalence of NCDs in Saudi, rather than assessing the inequities of NCDs services provision at the primary or other level of care [11–15]. Region of residence, education level, gender, and income are among the factors associated with inequities in NCDs disease burden. For diabetes, for example, the findings from a household survey analysis showed that diabetes prevalence was lower among highly educated people (OR: 0.328, 95% CI, 0.259-0.415) compared to people with education below a primary level [12]. A study carried out in Riyadh in 2021 indicated that the cost of health care services was the main out of pocket (OOP) expense for individuals with NCDs. Individuals with diabetes reported spending a median of SAR 501 (USD 134) per month. This highlights the financial burden that NCDs place on individuals and their families, and raises concerns regarding potential inequalities [16].

Other research has focused on accessibility to healthcare services, though not to NCD care, in particular. For instance, public primary care centers in Saudi vary widely in terms of capacity and distribution within and across urban and rural areas [7, 17]. More specifically, primary care centers in urban areas have more examination rooms but lower examination room densities, while the staffing density is higher in rural areas [17].

Few prior studies have assessed inequities in health seeking behaviors and uptake of screening services in Saudi [18, 19]. What has been published includes analysis of predictors of inequities related to the capacity and quality of primary care services. However, these studies did not specifically focus on NCD services access [20, 21]. Moreover, previous research often examined either a singular service or a specific demographic group, which cannot capture the broader landscape of primary care service utilization. For example, in a 2021 study based on 2018 Saudi Health Survey data, older age, higher education, insurance coverage, and being married were associated with more preventive health care check-ups [13]. Furthermore, a study that compared utilization of primary care services between five urban and five rural primary health care centers in Riyadh found that income and education are enabling factors to accessing healthcare, and, specifically, participants’ ability to pay for health insurance [22]. Similarly, the results from a study that included 2,786 Saudi women participating in a 2013 nationally representative Saudi Health Interview Survey assessed the socioeconomic inequalities in breast cancer screening [11]. It indicated that uptake of breast cancer screening and knowledge about breast cancer screening were higher among higher income women with more education [11].

This concept of unmet need has been widely used in Europe and Canada to complement conventional methods of measuring socioeconomic inequity using household surveys [23, 24]. It is defined as “the difference between services judged necessary to deal appropriately with health problems and services actually received” [25] and is used to assess health system performance and as a tool to assess the extent of healthcare access inequities [25]. For instance, it is measured in the Eurostat EU Statistics on Income and Living Conditions survey by asking whether there was a time in the previous 12 months when a respondent felt they needed medical care or dental care but did not receive it [26]. However, this measure depends on the person’s awareness of needs and willingness to report them. Other researchers have argued to use a more objective measure, one that refers to not receiving a service within a clinically acceptable interval [27]. It has been suggested that such an approach would limit bias in self-reporting based on socioeconomic status [28]. Previous studies have also attempted to generate novel approaches to measuring unmet need using household surveys [27–29].

In Saudi, little is known about the unmet need for NCDs preventive services and the factors contributing to variations in service utilization. Saudi has been committed to the sustainable development goals (SDGs), a core component of the United Nations General Assembly agenda to create a global development action plan. Target 3.8 of the SDGs includes universal health coverage (UHC) and equity [30]. Assessing the progress of UHC requires a measure to reflect the proportion of people who are unable to access the care they need.

Since the health transformation aims to contribute to the SDGs, gaining a better understanding of equity in healthcare utilization among those with NCDs represents an initial step towards improving equitable access to NCDs services [10]. To gain such an understanding, the objective of the present study was to quantify existing inequities in NCD treatment and preventive services utilization and associated regional and sociodemographic factors. By measuring objective unmet need as a proxy for NCDs services utilization, the study sought to establish a baseline for NCD services in the early stages of health system transformation implementation. We have focused on diabetes, hypertension, and dyslipidemia primary care services, along with breast and cervical cancer screenings, due to their increasing burden and as illustrative examples that highlight the unmet need for primary care services within the broader context of NCDs.

## Methods

### Study design

This study utilized data from the Kingdom of Saudi Arabia World Health Survey (KSAWHS 2019), containing nationally representative data of 10,000 households collected from Saudi’s 13 administrative regions. The main aim of the KSAWHS 2019 was to gather timely data on health-related indicators aligned with the SDGs and WHO standards. It includes demographic information, health insurance coverage, household wealth, health status, chronic conditions, healthcare utilization, reproductive health, family planning, violence against women, and child immunization. Additionally, the survey aims to estimate behavioral risk factors and the prevalence of conditions such as anaemia, hypertension, cholesterol levels, and diabetes mellitus among adults aged 15 and older. Two questionnaires were used for this survey: household and individual. They were based on the WHO’s World Health Survey and the Tunisian World Health Survey. The surveys reflect alignment with the SDGs and the list of the WHO’s 100 indicators. Further changes were made to be inclusive of national priorities. A panel of technical experts reviewed the questionnaires before they were translated to Arabic by a certified translator. The detailed methods of the survey can be found elsewhere [31].

The KSAWHS was implemented in 2019 by the Ministry of Health (MoH) in collaboration with the General Authority of Statistics (GASTAT) and the Saudi Health Council [31]. The Ministry of Health obtained ethical approval to conduct the survey from the General Directorate for Research and Studies at the MoH. Participation was voluntary and informed consent was obtained from all participants.

### Sample selection

The KSAWHS team started with a pre-test of 200 households as a convenience sample, with 15 households per region in the country. Data were collected by trained interviewees. The head of the household or the second most knowledgeable person available participated in the household questionnaire, while a randomly selected person 15 or older was administered the individual questionnaire through a face-to-face interview. Verbal or written consent was obtained from each respondent. Details on sampling techniques and quality measures are available [31]. The number of respondents in each region was weighted using standardized weights taking into account each stage selection and adjusted for non-response to produce a nationally representative sample. The response rate was 96.8% overall. To assess the outcome of primary care utilization among persons with NCDs, we identified individual questionnaire respondents who reported a diagnosis of diabetes, hypertension, or dyslipidemia as the three diseases of interest. For pap smear and mammography utilization outcomes, we restricted the sample to women above 21 years old and above 40, respectively.

### Outcome variables

The primary outcome was utilization of primary care services within the last 12 months among those who reported being diagnosed with an NCD. Respondents were asked whether they had visited a primary care physician in the public or the private sector in the past 12 months. Responses were categorized as Yes/No. Secondary outcomes of interest were the utilization of (1) cervical and (2) breast cancer screening services among screening eligible women. Women who were married, divorced, or widowed and above 21 years of age were asked if they had had a pap smear during their last pelvic exam. Women above age 30 years were asked if they had ever had a mammogram, but the analysis was restricted to women aged above 40 years for clinical relevance since the recommended age for women breast cancer screening in Saudi is above 40 years of age [32].

### Unmet need

For the purpose of this study, we relied on an objective unmet need definition. Unmet need is defined as reporting not seeing a primary care physician in the past 12 months among those who reported having a diagnosis of one of the NCDs of interest (diabetes, hypertension, and dyslipidemia). Unmet need among women who met the criteria of need for cervical and breast cancer screening was defined as never having cervical cancer screening and never having a mammography, respectively. We also considered subjective unmet need as one of the health services factors. This variable was created using the question that asked respondents whether they had received health care when needed in the past 12 months.

### Data analysis

Variables were selected based on linkages to the Kroger’s framework, which provides a holistic approach to analyzing and interpreting healthcare services utilization in developing countries. We also incorporated predictors of health services use and need (health outcomes) from the literature on) [33]. First, individual sociodemographic characteristics, including age, sex, marital status, education level, and employment were included. Nationality was dichotomized to Saudi and non-Saudi. Religion was also dichotomized to Muslims and non-Muslims.

Region of residence (urban/rural) and the administrative region were mapped to determine the variability of accessing services between the different administrative regions. Regions were aggregated into five major groups: “Central Region” includes Riyadh and Qassim, Eastern Province includes the “Eastern Region”, “Western Region” includes Makkah, Madinah and Bahah, “Southern Region” includes Asir, Najran, and Jizan, and the “Northern Region” includes Tabuk, Jawf, Northern Borders and Hail.

Monthly household income was categorized into quintiles: high income (above 15,000 SAR), upper- middle income (12,000 to 15,000 SAR), middle income (10,000 to 12,000 SAR), lower-middle income (6,000 to 10,000 SAR) and low income (below 6,000 SAR). The place of residence was categorized as rural or urban based on the General Authority of Statistics (GASTAT) classification of the corresponding enumeration area. Insurance variables varied depending on self-reported coverage. Public and private coverage were also assessed. Three insurance groups were created: those who were eligible for free government coverage, mandatory insurance for those covered by a private mandatory employer’s insurance, and voluntary insurance for those who paid OOP for private insurance.

The wealth index is a composite measure developed by the Demographic and Health Surveys (DHS) program to evaluate a household’s overall living standard [34]. The survey team used Principal Component Analysis (PCA) to generate a continuous scale of household wealth using a collection of household indicators such as house building materials, water and sanitation facilities, and household ownership of assets (e.g., televisions and refrigerators). We disaggregated the scale into five wealth quintiles ranging from the 1st quintile (lowest/poorest) to the 5th quintile (highest/wealthiest) [34].

### Statistical analysis

We analyzed the demographic characteristics of the study population, overall and stratified by regions. A multivariable logistic regression model was used to identify significant predictors of utilization of primary care services for prevention and treatment of NCDs, and for unmet need. We estimated separate regression models to identify significant factors affecting each of the secondary outcomes related to breast and cervical cancer screenings. Each model controlled for age, sex, religion, nationality, education, area of living, region, employment, marital status, income levels, wealth index, insurance coverage, and perceived healthcare need. Because primary care services were more readily available through the public sector and were limited in the private sector during the time of data collection, we only controlled for government coverage in the first model (objective unmet need of NCDs). We included both government and mandatory insurance coverage in the other models.

For each regression, we accounted for clustering of standard errors within regions, to confirm that results were not driven simply by between-region variation. The significant factors from each model analysis were identified using a 0.05 significance level. Here we report adjusted odds ratios with 95% confidence intervals for variables in the final model. All analyses were performed in R Studio [35] taking into account complex sampling design and weighting.

## Results

### Summary statistics

**Fig. 1:**
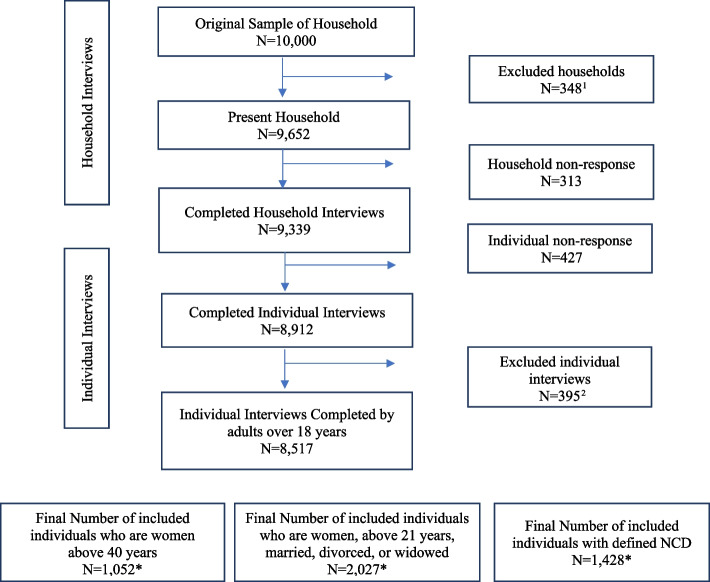
Number of completed household and individual interviews included in the analysis. 1Excluded based on the following interview result categories: household absent for extended period of time; Dwelling vacant; Address not a dwelling; Dwelling destroyed; Dwelling under construction; Dwelling status unknown. 2 Excluded for the following reasons: 395 completed interviews were excluded because they were completed by individuals under the age of 18 years. ^*^Outcome non-response item excluded

Out of the 10,000 sampled households, 9,652 were occupied by one or more individuals, and 9,339 respondents completed a household questionnaire (household response rate = 96.8%). A total of 8,912 respondents completed an individual questionnaire (individual response rate of 95.4%). The final dataset excluded data from respondents aged under 18 years, leading to a final sample of 8,517 respondents (Fig. 1). See Appendix 1 for details on the weighted demographic characteristics of the full sample of the respondents. The final dataset excluded respondents who had missing outcomes.

Table 1 shows the weighted demographic characteristics for the three sub-populations included in this analysis. Among the 1,428 people who reported having an NCD diagnosis (diabetes, hypertension, and dyslipidemia), 46% were females, and 59% were above 45 years of age. The majority (64.3%) was below the age of 55 years while persons above age 65 years comprised 17% of the sample. A plurality of respondents was from the western regions and central regions (both at 34%), followed by the southern and Eastern regions (14% and 13%), respectively. Most respondents in the questionnaire were married (72%). The respondents were largely Saudi citizens (90%); non-Saudis represented only10% of the sample. Just under half (46%) of the sample reported being employed and more than 44% had higher than a secondary level of education. Most respondents resided in urban areas (82%). Just over one-third (39.4%) were middle-income, while just under one-third (31%) were high-income. The majority of respondents (92%) were covered by free health care services; 21% had mandatory insurance. The majority of respondents (92%) qualified for free governmental services, while only 21% had essential insurance coverage, and 3.5% had private insurance. Thirty five percent reported a perceived need for healthcare within the last 12 months.

**Table 1:** Characteristics of analysis populations

	Individuals who reported an NCDs	Women who are eligible for mammogram^b^	Women who are eligible for pap smear^c^
Characteristic	*N* = 1,428^a^	*N* = 1,052^a^	*N* = 2,027^a^
**Sex**			
Male	773 (54%)	0 (0%)	0 (0%)
Female	655 (46%)	1,052 (100%)	2,027 (100%)
**Age categories**			
18-24 years	61 (4.3%)	0 (0%)	157 (7.8%)
25-34 years	207 (15%)	0 (0%)	869 (43%)
35-44 years	331 (23%)	261 (25%)	486 (24%)
45-54 years	309 (22%)	397 (38%)	259 (13%)
55-64 years	283 (20%)	232 (22%)	153 (7.6%)
+65 years	236 (17%)	162 (15%)	102 (5.0%)
**Region**			
Central	485 (34%)	222 (21%)	662 (33%)
Eastern	186 (13%)	167 (16%)	337 (17%)
Western	483 (34%)	426 (40%)	526 (26%)
Southern	202 (14%)	165 (16%)	337 (17%)
Northern	72 (5.1%)	72 (6.9%)	164 (8.1%)
**Marital status**			
Never married	108 (7.6%)	0 (0%)	0 (0%)
Currently married	1,034 (72%)	736 (70%)	1,681 (83%)
Separated/ Divorced	105 (7.3%)	84 (8.0%)	158 (7.8%)
Widowed	181 (13%)	232 (22%)	188 (9.3%)
**Household Wealth index** ^d^			
Lowest quintile	236 (17%)	194 (18%)	376 (19%)
Second quintile	299 (21%)	212 (20%)	477 (24%)
Middle quintile	222 (16%)	185 (18%)	426 (21%)
Fourth quintile	288 (20%)	234 (22%)	428 (21%)
Highest quintile	382 (27%)	226 (21%)	320 (16%)
**Household Income categories** ^e^			
Low income	413 (30%)	392 (38%)	537 (27%)
Lower-middle income	196 (14%)	133 (13%)	384 (19%)
Middle income	130 (9.4%)	87 (8.5%)	233 (12%)
Upper-middle income	218 (16%)	159 (16%)	408 (21%)
High income	434 (31%)	252 (25%)	420 (21%)
**Nationality**			
Saudi	1,284 (90%)	950 (90%)	1,817 (90%)
Non-Saudi	144 (10%)	102 (9.7%)	210 (10%)
**Education level**			
No formal education	170 (12%)	208 (20%)	136 (6.7%)
Less than secondary	310 (22%)	344 (33%)	363 (18%)
Secondary	320 (22%)	243 (23%)	645 (32%)
More than secondary	626 (44%)	255 (24%)	884 (44%)
**Employment**			
Yes	669 (47%)	192 (18%)	545 (27%)
No	391 (27%)	228 (22%)	477 (24%)
Never worked before	368 (26%)	632 (60%)	1,005 (50%)
**Type of place of residence**			
Urban	1,174 (82%)	872 (83%)	1,632 (81%)
Rural	253 (18%)	181 (17%)	395 (19%)
**Religion**			
Muslim	1,424 (100%)	1,051 (100%)	2,023 (100%)
Non-Muslim	4 (0.3%)	1 (0.1%)	4 (0.2%)
**Eligibility for government free services**			
Yes	1,291 (92%)	965 (92%)	1,861 (92%)
No	113 (8.1%)	80 (7.6%)	166 (8.2%)
**Health insurance coverage**			
Yes	293 (21%)	110 (10%)	292 (14%)
No	1,135 (79%)	941 (90%)	1,733 (86%)
**Private insurance coverage**			
Yes	51 (3.5%)	43 (4.1%)	54 (2.7%)
No	1,377 (96%)	1,009 (96%)	1,972 (97%)
**Subjective health care need**	495 (35%)	228 (23%)	463 (23%)

^a^n (%)

^b^Women who are 40 years and above

^c^Women 21 years and above who are married, divorced and widowed

^d^Composite measure calculated using data about ownership of consumer material such television and cars, household characteristics such as building material, source of drinking water, toilet facilities and other characteristics relevant to wealth status

^e^Variable has missing data

Among the 1,052 women eligible for breast cancer screening, 60% were 45 to 64 years of age, and 33% had below a secondary education level. The majority had never been employed (60%). Nearly half (44%) of women who were eligible for a pap smear (*n*=2,027) had more than a secondary level of education. Among women above the age of 40, 37.5% were from a middle-income household, whereas among women eligible for a pap smear, 52% were from a middle-income household. Only 23% of both groups (those eligible for breast cancer screening and those eligible for a pap smear) reported a subjective health care need within the last 12 months.

Of those with a diagnosed NCD, 35.4% had not seen a general practitioner (GP) in the last 12 months, so had an unmet need (Table 2). Only 17.89% of women above 40 years of age had ever had a mammogram, while only 20% of women above 21 years of age and who were married, widowed, or divorced had had a cervical cancer screening during the last pelvic examination (Table 2).

**Table 2:** Outcome summary statistics

Variable	n	%	SE	CI
*Utilization of Primary Preventive Services*				
Patients diagnosed with one or more 1 NCDs who saw a GP in the past 12 months (Objective met need) *N*=1,428				
Yes	921	51.31%	0.02	0.61, 0.68
No	507	32.33%	0.02	0.32, 0.39
*Cancer Screening*				
Pap smear test at the last pelvic examination *N*=2,027				
Yes	399	19.70%	0.01	0.17, 0.22
No	1628	80.30%	0.01	0.78, 0.83
Ever had a mammogram *N*=1,052				
Yes	188	17.89%	0.02	0.15, 0.21
No	864	82.11%	0.02	0.79, 0.85

### Predictors of unmet need among people with reported NCD diagnosis

**Fig. 2:**
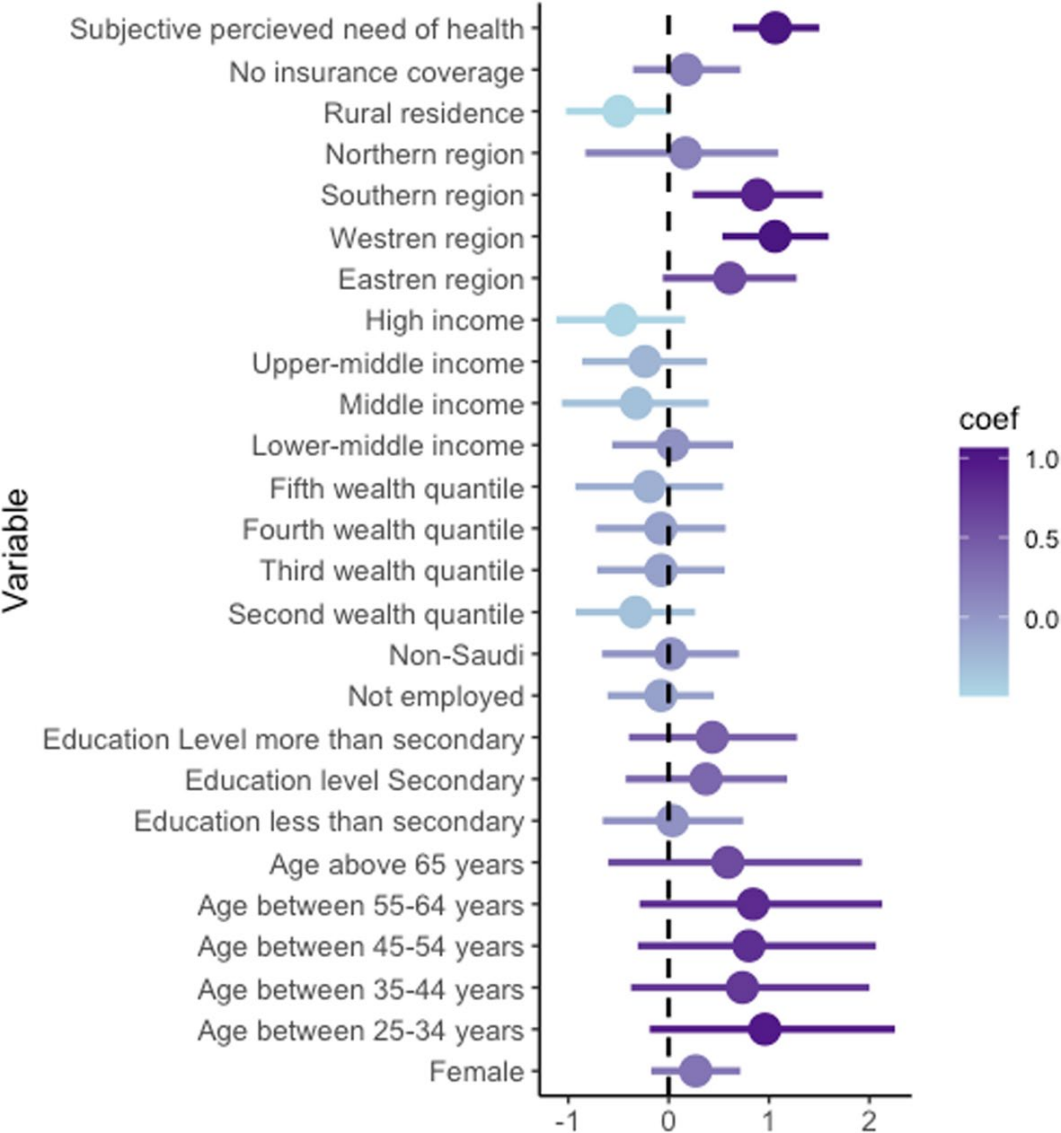
Forest plot of objective unmet need regression model coefficient among people who reported a non-communicable disease (NCD) diagnosis

In this logistic regression analysis (Table 3), we found that several factors were significantly associated with the likelihood of having an unmet need among those who reported having an NCD diagnosis. Among individuals who reported being diagnosed with an NCD, those who were more affluent had a higher probability of having a met need related to receiving NCDs services in primary care. Living in the Western (OR 2.86, *p* < 0.001), Southern (OR 2.16, *p*=0.016), or Eastern region (OR 1.86, *p*=0.058) was associated with a higher likelihood of having an unmet need among people with a reported NCD in comparison to the central regions. Adults with a known NCD diagnosis residing in rural areas were less likely to have an unmet need (visiting a GP within the last 12 months) compared to those residing in urban areas (OR 0.58, *p*=0.029). Respondents who did not have a perception of needing healthcare within the last 12 months were three times as likely to have an unmet need compared to those who perceived a need for healthcare (OR 3.00, *p*<0.001). No other variables were significantly associated with having an objective unmet need. (Figure 2) shows the regression model coefficient forest plot.

**Table 3:** Factors associated with not having a GP visit within the past 12 months in patients diagnosed with at least one NCD

Characteristic	OR^a^	95% CI^a^	*p*-value
**Sex**			
*Male*	—	—	
*Female*	1.36	0.94, 1.97	0.11
**Age categories**			
*18-24 years*	—	—	
*25-34 years*	3.15	0.93, 10.7	**0.066**
*35-44 years*	2.54	0.75, 8.60	0.13
*44-54 years*	2.75	0.80, 9.42	0.11
*55-64 years*	2.92	0.83, 10.3	0.10
*+65 years*	2.29	0.61, 8.56	0.2
**Education level**			
*No formal education*	—	—	
*Less than secondary*	1.04	0.58, 1.86	>0.9
*Secondary*	1.53	0.78, 2.99	0.2
*More than secondary*	1.63	0.81, 3.27	0.2
**Employment**			
*Yes*	—	—	
*No*	0.90	0.58, 1.41	0.7
**Nationality**			
*Saudi*	—	—	
*Non-Saudi*	1.03	0.59, 1.79	>0.9
**Household Wealth index** ^b^			
Lowest quintile	—	—	
Second quintile	0.73	0.45, 1.16	0.2
Middle quintile	0.92	0.55, 1.55	0.8
Fourth quintile	0.93	0.54, 1.61	0.8
Highest quintile	0.81	0.40, 1.62	0.6
**Household Income categories**			
*Low income*	—	—	
*Lower-middle income*	1.04	0.65, 1.67	0.9
*Middle income*	0.72	0.41, 1.26	0.2
*Upper-middle income*	0.78	0.45, 1.35	0.4
*High income*	0.60	0.34, 1.07	**0.082**
**Region**			
*Central*	—	—	
*Eastern*	1.86	0.98, 3.52	**0.058**
*Western*	2.90	1.74, 4.83	**<0.001**
*Southern*	2.49	1.40, 4.44	**0.002**
*Northern*	1.20	0.65, 2.19	0.6
**Type of place of residence**			
*Urban*	—	—	
*Rural*	0.58	0.35, 0.95	**0.029**
**Health insurance coverage**			
*Yes*	—	—	
*No*	1.18	0.75, 1.86	0.5
**Subjective health care need**			
*Yes*	—	—	
*No*	3.00	2.06, 4.35	**<0.001**

^a^*OR* Odds Ratio, *CI* Confidence Interval

^b^Composite measure calculated using data about ownership of consumer material such television and cars, household characteristics such as building material, source of drinking water, toilet facilities and other characteristics relevant to wealth status

### Predictors of utilizations of breast and cervical cancer screening services

Women who were between 35-44 years, 55-64 years, and 65 years or above were less likely to undergo a cervical cancer screening in comparison to women in younger age groups (OR 0.52, 0.34 and 0.11). Meanwhile, being a widow was strongly associated with a higher probability of having been screened for cervical cancer and breast cancer (OR 3.11, *p*<0.001) and (OR 2.38, *p*=0.005), respectively. Moreover, education level was a significant predictor for cancer screening. Women who had a secondary level education had four times the odds of being screened for breast cancer (OR 4.25, *p*=0.025), and five times the odds of undergoing cervical cancer screening at their last pelvic exam (OR 5.07, *P*=0.012). Similarly, having a secondary education (OR 2.24, *P*=0.036) or higher than secondary education level (OR 2.72, *p*=0.02) was associated with higher chances of ever having a mammogram. Employment and nationality seemed to have no influence on both outcomes when controlling for other variables. Although non-Saudis had twice the odds of ever having a mammogram, the difference was not significant (OR 2.36, *p*=0.072).

Additionally, while the results for having a mammogram were not statistically significant at the 0.05 level (OR 2.23, *p*=0.081), wealth emerged as another strong predictor of cervical and breast cancer screening. Women in the highest wealth quintile of households were twice as likely both to be screened for HPV (OR 1.92, *P*=0.026) and to have had a mammogram. Likewise, a strong effect was observed for household income. Coming from a middle (OR 1.99, *P*=0.026), upper middle (OR 3.47, *p*<0.001), or high income household (OR 2.59, *p*<0.001) increased the probability of having had cervical cancer screening. A similar association, though with less statistical significance, applied to respondents in high income households regarding breast cancer screening, when compared to those in low-income households (OR 1.81, *p*=0.069). Conversely, coming from a lower middle-income household was associated with a lower probability of ever having a mammogram (OR 0.38, *p*=0.054).

Regional location emerged as an important factor in access to cervical and breast cancer screening. Women living in Southern regions were 75% less likely to be screened for cervical cancer in comparison to those in central regions. The Eastern region was another region where women were less likely to be screened for cervical cancer, but the difference was not significant. Respondents in rural areas had twice the chance of having had a mammogram or a pap test in comparison to respondents in urban areas (OR 2.41, *p*=0.003) and (OR 2.07, *P*=0.001), respectively.

**Fig. 3:**
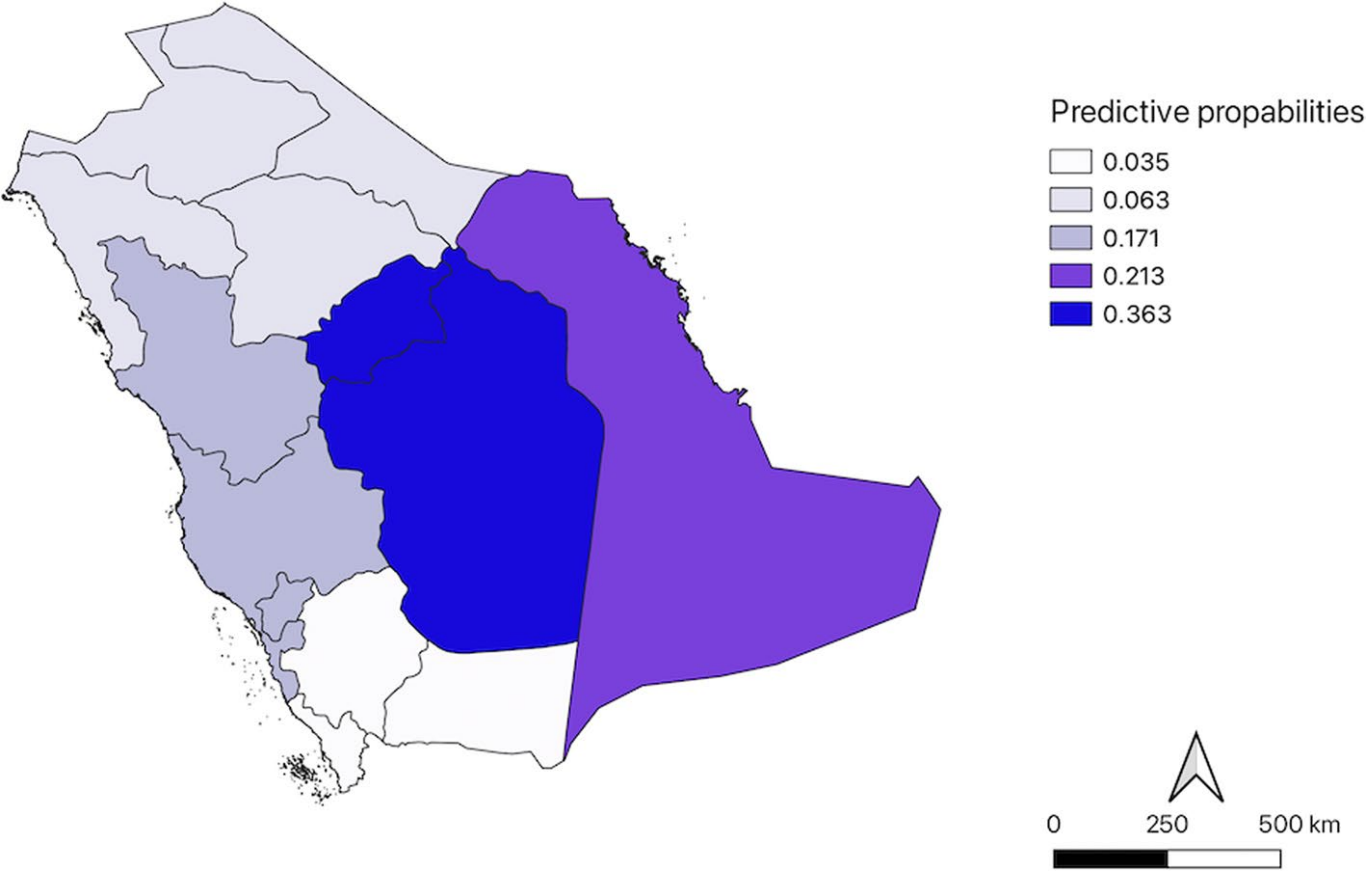
A regional analysis of mammography utilization: Mapping the probability of ever having a mammogram

Figure 3 shows the predicted probability of women ever having a mammogram in different regions. Women in the Central region had the highest predicted probability (0.36), followed by the Eastern region (0.21), and the Western region (0.17). The Southern and Northern regions had the lowest probability (0.03 and 0.06, respectively) of having had a mammogram.

A perceived need for healthcare was a significant factor influencing the probability of a woman’s cancer screening utilization. Women who did not have a perception of needing healthcare during a calendar year were 45% less likely to be screened for a pap test and 60% less likely to ever have had a mammogram in comparison to those with a perceived need for healthcare (Tables 4, 5).

**Table 4:** Factors associated with having a pap test during the last pelvic exam for women above 21 years

Characteristic	OR^a^	95% CI^a^	*p*-value
**Age categories**			
*18-24 years*	—	—	
*25-34 years*	0.68	0.39, 1.16	0.2
*35-44 years*	0.52	0.29, 0.93	**0.028**
*45-54 years*	1.24	0.66, 2.34	0.5
*55-64 years*	0.34	0.13, 0.90	**0.029**
*+65 years*	0.11	0.03, 0.42	**0.001**
**Marital status**			
*Currently married*	—	—	
*Currently married*	—	—	
*Separated/ Divorced*	1.08	0.65, 1.79	0.8
*Widowed*	3.11	1.59, 6.06	**<0.001**
**Education level**			
*No formal education*	—	—	
*Less than secondary*	2.30	0.71, 7.45	0.2
*Secondary*	4.25	1.20, 15.0	**0.025**
*More than secondary*	5.07	1.42, 18.1	**0.012**
**Employment**			
*Yes*	—	—	
*No*	1.03	0.71, 1.49	0.9
**Nationality**			
*Saudi*	—	—	
*Non-Saudi*	1.25	0.67, 2.31	0.5
**Household Wealth index **^b^			
Lowest quintile	—	—	
Second quintile	1.60	0.96, 2.65	0.071
Middle quintile	1.13	0.66, 1.95	0.7
Fourth quintile	1.50	0.88, 2.56	0.14
Highest quintile	1.92	1.08, 3.40	**0.026**
**Household Income categories**			
*Low income*	—	—	
*Lower-middle income*	1.09	0.62, 1.91	0.8
*Middle income*	1.99	1.09, 3.64	**0.026**
*Upper-middle income*	3.47	2.05, 5.87	**<0.001**
*High income*	2.59	1.46, 4.57	**0.001**
**Region**			
*Central*	—	—	
*Eastern*	0.60	0.35, 1.03	0.065
*Western*	0.83	0.50, 1.36	0.5
*Southern*	0.24	0.13, 0.43	**<0.001**
*Northern*	0.70	0.37, 1.33	0.3
**Type of place of residence**			
*Urban*	—	—	
*Rural*	2.07	1.33, 3.22	**0.001**
**Eligibility for free government services**			
*Yes*	—	—	
*No*	1.79	0.85, 3.76	0.13
**Health insurance coverage**			
*Yes*	—	—	
*No*	0.97	0.61, 1.54	0.9
**Subjective health care need**			
*Yes*	—	—	
*No*	0.55	0.39, 0.79	**0.001**

^a^*OR *Odds Ratio, *CI* Confidence Interval

^b^Composite measure calculated using data about ownership of consumer material such television and cars, household characteristics such as building material, source of drinking water, toilet facilities and other characteristics relevant to wealth status

**Table 5:** Factors associated with ever having had a mammogram among women above 40 years

Characteristic	OR^a^	95% CI^a^	*p*-value
**Age categories**			
*35-44 years*	—	—	
*45-54 years*	1.52	0.87, 2.65	0.14
*55-64 years*	1.72	0.85, 3.51	0.13
*+65 years*	1.17	0.50, 2.71	0.7
**Marital status**			
*Currently married*	—	—	
*Currently married*	—	—	
*Separated/ Divorced*	1.75	0.69, 4.46	0.2
*Widowed*	2.38	1.31, 4.33	**0.005**
**Education level**			
*No formal education*	—	—	
*Less than secondary*	1.74	0.90, 3.37	**0.10**
*Secondary*	2.24	1.05, 4.77	**0.036**
*More than secondary*	2.72	1.17, 6.31	**0.020**
**Employment**			
*Yes*	—	—	
*No*	1.57	0.87, 2.82	0.13
**Nationality**			
*Saudi*	—	—	
*Non-Saudi*	2.36	0.93, 6.03	0.072
**Household Wealth index** ^b^			
Lowest quintile	—	—	
Second quintile	1.22	0.53, 2.78	0.6
Middle quintile	0.76	0.29, 2.01	0.6
Fourth quintile	1.12	0.47, 2.70	0.8
Highest quintile	2.23	0.91, 5.50	0.081
**Household Income categories**			
*Low income*	—	—	
*Lower-middle income*	0.38	0.14, 1.02	**0.054**
*Middle income*	0.71	0.27, 1.83	0.5
*Upper-middle income*	1.24	0.65, 2.37	0.5
*High income*	1.81	0.95, 3.44	**0.069**
**Region**			
*Central*	—	—	
*Eastern*	0.95	0.48, 1.89	0.9
*Western*	0.52	0.28, 0.99	**0.047**
*Southern*	0.09	0.03, 0.30	**<0.001**
*Northern*	0.17	0.08, 0.39	**<0.001**
**Type of place of residence**			
*Urban*	—	—	
*Rural*	2.41	1.36, 4.27	**0.003**
**Eligibility for free government services**			
*Yes*	—	—	
*No*	0.33	0.09, 1.22	0.10
**Health insurance coverage**			
*Yes*	—	—	
*No*	1.31	0.75, 2.29	0.3
**Subjective health care need**			
*Yes*	—	—	
*No*	0.40	0.26, 0.63	**<0.001**

^a^*OR* Odds Ratio, *CI* Confidence Interval

^b^Composite measure calculated using data about ownership of consumer material such television and cars, household characteristics such as building material, source of drinking water, toilet facilities and other characteristics relevant to wealth status

## Discussion

In this cross-sectional analysis of data from the 2019 KSAWHS, we highlight the unmet need across a range of important outcomes. Our results play a critical role as proving baseline for Saudi’s health transformation efforts. We found that more than 35% of people with diagnosed diabetes, hypertension, or dyslipidemia had an unmet need for primary care services. This means that one of three patients with a NCD appears to lacking preventive care for a diagnosed NCD. Only 20% of surveyed women had had a cervical cancer screening during their last pelvic exam, while only 18% of eligible women had ever had a mammogram. To our knowledge, this is the one of a handful of studies of unmet need on a large scale of the population [36–38]. Using a nationally representative survey, we identified critical factors associated with NCD treatment and preventative services utilization, as well as regional variation in use of primary care services for NCD care and women’s cancer screening services, topics largely underexplored in the literature in the Saudi context.

Our finding of low utilization of cancer screening services is consistent with previous studies that estimated the utilization of breast cancer and cervical cancer screening in Gulf Countries from 2014 data. Receipt of breast cancer screening within the last two years among women between 40-74 years of age was estimated at 4.9%, far lower than in neighboring gulf countries like Oman (8.9%) [39]. Our analysis found that 7.6% of women in Saudi had had a pap smear test at their last pelvic exam, compared to 10.6% in Oman, 17.7% in Kuwait, and 28.0% in the United Arab Emirates [39].

Apart from large unmet needs in the detection and treatment of NCDs, there was regional variability in the levels of unmet need among those who reported having an NCD diagnosis. Two Geographic Information System studies in Saudi Arabia found issues related to geographic access to healthcare facilities in Western and Southern regions [40, 41]. This variability might be explained by particular differences in primary care infrastructure and resources allocation across regions, resulting in variation in services that are provided, and, consequently, in disparities in health outcomes [17]. These finding are consistent with evidence on the geographical heterogeneity of unmet need in publicly funded systems such as Italy and Thailand [42, 43]. Women living in Saudi’s Southern regions are significantly less likely to be screened for cervical and breast cancer. In Southern regions such as Jazan and Aseer, religious and traditional beliefs, combined with poor knowledge related to health, are among the factors that may influence pap smear utilization [44, 45]. Similar factors might also explain what we found in Northern regions, where women experienced a lower likelihood of utilizing breast cancer screening.

Although resources have been allocated toward breast cancer screening through the national public health initiative and other regional initiatives, varying degrees of screening uptake are clear across regions in Saudi Arabia [46–48]. Methods of breast cancer screening delivery are also variable across regions. For instance, mammography mobile clinics are more available in rural areas [46–48]. Primary care physicians typically refer women to higher level centers to be screened, while some regions rely on mobile screening clinics. Other areas, such as the Eastern region, have stand-alone mammography centers [46, 49]. Other factors strongly associated with women’s cancer screening services utilization included education, wealth, and income levels, a finding consistent with prior research in Saudi [50, 51].

Furthermore, stark inequities related to urbanization emerged from our analysis. Surprisingly, in our study we found that populations residing in rural areas were less likely to have unmet needs. This finding is inconsistent with other international and national studies that report poorer access and utilization of primary health care centers (PHCs) in rural areas [22, 52] but consistent with findings of a recently published study that assessed dental utilization using the KSAWHS [38]. A possible explanation for this finding is the difference between the availability of health care facilities. Populations in rural areas depend on public facilities due to a lack of private sector facilities. Although facilities available in rural areas have fewer resources, care is less fragmented and may be more reliable. Another possible explanation might be the cultural differences between urban and rural areas, which may affect health care seeking behavior. For instance, there may be higher trust in the public system given fewer healthcare options, as well as stronger doctor-patient relationships and better continuity of care through available PHCs in rural areas [53, 54]. We recommend that future studies explore this issue further. Additionally, these results are sensitive to the definition of urban areas used in the KSAWHS; GASTAT defines urban areas by population size of more than 100,000 people [55]. Populations in rural areas represented 18% of this survey sample and are known to have lower NCD burden in comparison to urban areas. To further enhance the ongoing efforts towards UHC, we encourage those working in health system reform to pay close attention to technical definitions (such as urban versus rural) and the terms that underpin the data used in decision-making.

Our study provides important insights into the factors associated with objective unmet health care needs. We found that perceived health care need was the most significant predictor of low unmet need among people with NCDs. Perceived need is a subjective measure that is different from clinically assessed objective need [56] Probing deeper into reported reasons of unmet need and available clinical data in future studies may highlight equity implications that may be taken into account in ongoing efforts to enhance the system’s responsiveness, such as cost barriers and providers’ availability.

Our results reveal an unmet need for NCDs-related services nationally, particularly among specific demographic groups. However, the limitations of the existing dataset precluded a comprehensive analysis of NCD-related inequities and their root causes. Therefore, to conduct a comprehensive assessment that can play a role in policy-making to improve equity in health outcomes, it is essential to analyze the impact of diverse equity parameters (e.g., sex, nationality, region of residence) on health outcomes. Such examinations would provide the information decision-makers require to effectively incorporate these factors into the decision-making process.

Even though Saudi’s healthcare system at the time of the survey provided free access to primary care services for eligible citizens and groups of eligible expatriates, our results raise an important question regarding future steps for how equity considerations are included in prioritization activities and resources allocation in Saudi’s planned health financial transformation [57]. A study from Thailand that underwent a health reform with a commitment to UHC found that prioritizing tertiary care without adequate investment in primary care can exacerbate existing inequities [58, 59] In Saudi Arabia, the recent changes in the essential benefit package of private insurance to include an annual pap test is an example of policy changes that are required to reduce equity gaps. Entities responsible for health provision in each region are encouraged to measure inequities in healthcare access and prioritize this information when allocating resources. Further exploration of equity-related factors during the ongoing transformation will be essential to achieve UHC and improved service utilization [59] A relevant lesson could be learned from Costa Rica, where integrating a monitoring mechanism of equity parameters contributed to a more equitable primary care [60].

At this critical phase of the transformation, we recommend that policymakers and health system reformers, particularly those engaged in the development of national health insurance programs, adopt an equity-based approach and customize programs to address the requirements of both the general population and vulnerable population groups. Such a strategy can help ensure that disparities in NCD services are addressed, particularly among marginalized communities. In the context of implementing model of care initiatives, it is essential to prioritize the implementation of equitable measures and evaluate the effectiveness of existing programs, including breast cancer awareness campaigns, other NCD initiatives, and newly introduced NCD services initiatives. Such measures will enable program expansion in a cost-effective manner, thereby increasing the reach of these initiatives to a broader population.

Our study has multiple strengths. First, it is the first study to look into regional variation in primary care services use among people with NCDs and to explore pap smear uptake variation between regions in Saudi Arabia. Second, the study followed a pragmatic approach, using a tailored framework to analyze factors influencing services utilization in developing countries [33]. It also contributes to existing evidence that can be used as a baseline for future NCDs services and cancer screening equity studies.

The study also has some limitations. Saudis represented 87% of the population in the survey sample of 10,000 households while non-Saudis represented only 13%. This is different from the general population distribution, whereby expatriates represent one third of Saudi’s total population [61]. This study did not consider all the factors that might influence health services utilization, such as social beliefs, health facilities distribution, OOP spending, and other structural factors. This cross-sectional study used a crude outcome measure of primary care visits as a proxy for NCDs preventive and treatment services utilization, in addition to relying on self-reported NCD diagnosis, which may have systematic bias. Also, the true regional variation might be masked due to the way that data in regions are aggregated. Finally, the survey excluded single non-married women from the pap smear question, which means that the results are missing some clinically-eligible women. It is important to note that the findings are associational, and causality cannot be determined based on these findings.

## Conclusion

This study highlights disparities of primary care services utilization by region, population density, wealth, income, and education. Because utilization is related to perceptions of need, this study highlights the importance of conducting targeted awareness campaigns at the regional level to enhance both perceived needs for, as well as ultimate utilization of, healthcare services for a range of services, including NCDs. While our research took initial steps to quantify unmet need related to receiving NCD primary care, further research is required to better understand the extent of unmet primary care needs for NCDs, and effective ways to address the underlying contributing factors to achieve the health transformation goals.

### Supplementary Information

**Supplementary Material 1.** 

## Data Availability

The data supporting the findings of our study originate from the Saudi Arabian Ministry of Health. These data were utilized under specific license terms for the current study, and as such, they are not publicly accessible. Interested parties can request access to the data from the authors, subject to approval and in accordance with the policies set forth by the Saudi Arabian Ministry of Health Data Governance Office. Requests for data access should be directed to Data-office@moh.gov.sa and will be considered on a reasonable basis upon permission from the Ministry of Health Data Governance Office.

## Abbreviations

CVDs: Cardiovascular diseases
DHS: Demographic and Health Surveys
DALYs: Disability-adjusted life years
GASTAT: General Authority of Statistics
IHME: Institute for Health Metrics and Evaluation
KSAWHS: Kingdom of Saudi Arabia World Health Survey
LDL: Low density lipoprotein
MoH: Ministry of Health
NCDs: Non-communicable diseases
OOP: Out of pocket
PHCs: Primary health care centers
SDGs: Sustainable development goals
UHC: Universal health coverage
