# Supplementary material for: Equity and unmet need of non-communicable diseases services in Saudi Arabia using a National Household Survey (2019)
Source: BMC Health Serv Res. 2024 Mar 16;24:346. doi: 10.1186/s12913-024-10787-6 (PMC10943914; doi:10.1186/s12913-024-10787-6)
Supplement: Supplementary file 1 — Supplementary Material 1. [file 12913_2024_10787_MOESM1_ESM.docx]

**Supplementary Table1: Sociodemographic characteristics of survey respondents**

| **Characteristic** | **Overall,**  **N = 8,517^1^** | **Central,**  **N = 2,625^1^** | **Eastern,**  **N = 1,089^1^** | **Western, N = 2,898^1^** | **Southern, N = 1,203^1^** | **Northern, N = 702^1^** | **p-value^2^** |
| --- | --- | --- | --- | --- | --- | --- | --- |
| **Sex** |  |  |  |  |  |  | <0.001 |
| Male | 4,502 (53%) | 1,488 (57%) | 516 (47%) | 1,446 (50%) | 661 (55%) | 392 (56%) |  |
| Female | 4,015 (47%) | 1,137 (43%) | 573 (53%) | 1,452 (50%) | 543 (45%) | 310 (44%) |  |
| **Age categories** |  |  |  |  |  |  | <0.001 |
| 18-24 years | 1,413 (17%) | 432 (16%) | 143 (13%) | 589 (20%) | 164 (14%) | 85 (12%) |  |
| 25-34 years | 3,285 (39%) | 1,191 (45%) | 348 (32%) | 996 (34%) | 461 (38%) | 290 (41%) |  |
| 35-44 years | 2,014 (24%) | 581 (22%) | 333 (31%) | 582 (20%) | 311 (26%) | 207 (30%) |  |
| 45-54 years | 946 (11%) | 245 (9.3%) | 133 (12%) | 368 (13%) | 123 (10%) | 77 (11%) |  |
| 55-64 years | 524 (6.1%) | 103 (3.9%) | 83 (7.6%) | 234 (8.1%) | 82 (6.8%) | 22 (3.1%) |  |
| +65 years | 334 (3.9%) | 73 (2.8%) | 50 (4.6%) | 129 (4.5%) | 63 (5.2%) | 20 (2.9%) |  |
| **Marital status** |  |  |  |  |  |  | <0.001 |
| Never married | 1,884 (22%) | 670 (26%) | 174 (16%) | 717 (25%) | 211 (18%) | 112 (16%) |  |
| Currently married | 5,954 (70%) | 1,686 (64%) | 849 (78%) | 1,955 (67%) | 907 (75%) | 558 (79%) |  |
| Separated/ Divorced | 343 (4.0%) | 150 (5.7%) | 30 (2.8%) | 109 (3.8%) | 37 (3.1%) | 17 (2.4%) |  |
| Widowed | 336 (4.0%) | 118 (4.5%) | 37 (3.4%) | 117 (4.0%) | 49 (4.1%) | 16 (2.2%) |  |
| **Wealth index** |  |  |  |  |  |  | <0.001 |
| 1 | 2,096 (25%) | 372 (14%) | 257 (24%) | 912 (31%) | 388 (32%) | 166 (24%) |  |
| 2 | 1,857 (22%) | 521 (20%) | 234 (22%) | 649 (22%) | 314 (26%) | 140 (20%) |  |
| 3 | 1,624 (19%) | 429 (16%) | 233 (21%) | 573 (20%) | 238 (20%) | 152 (22%) |  |
| 4 | 1,528 (18%) | 558 (21%) | 205 (19%) | 434 (15%) | 172 (14%) | 159 (23%) |  |
| 5 | 1,412 (17%) | 745 (28%) | 160 (15%) | 330 (11%) | 92 (7.6%) | 85 (12%) |  |
| **Income categories** |  |  |  |  |  |  | <0.001 |
| Low income | 2,383 (29%) | 496 (19%) | 308 (30%) | 964 (35%) | 428 (36%) | 186 (27%) |  |
| Lower-middle income | 1,623 (20%) | 470 (18%) | 271 (26%) | 475 (17%) | 225 (19%) | 183 (27%) |  |
| Middle income | 929 (11%) | 261 (10%) | 143 (14%) | 317 (11%) | 111 (9.4%) | 97 (14%) |  |
| Upper-middle income | 1,571 (19%) | 570 (22%) | 168 (16%) | 488 (17%) | 222 (19%) | 122 (18%) |  |
| High income | 1,740 (21%) | 762 (30%) | 142 (14%) | 547 (20%) | 193 (16%) | 95 (14%) |  |
| **Nationality** |  |  |  |  |  |  | <0.001 |
| Saudi | 7,419 (87%) | 2,294 (87%) | 1,055 (97%) | 2,365 (82%) | 1,070 (89%) | 635 (91%) |  |
| Non-Saudi | 1,097 (13%) | 331 (13%) | 34 (3.1%) | 533 (18%) | 133 (11%) | 66 (9.4%) |  |
| **Education level** |  |  |  |  |  |  | <0.001 |
| No formal education | 346 (4.1%) | 60 (2.3%) | 48 (4.4%) | 109 (3.8%) | 105 (8.7%) | 23 (3.3%) |  |
| Less than secondary | 1,173 (14%) | 257 (9.8%) | 184 (17%) | 391 (13%) | 208 (17%) | 132 (19%) |  |
| Secondary | 2,913 (34%) | 812 (31%) | 479 (44%) | 960 (33%) | 411 (34%) | 252 (36%) |  |
| More than secondary | 4,079 (48%) | 1,491 (57%) | 377 (35%) | 1,438 (50%) | 479 (40%) | 294 (42%) |  |
| **Employment** | 4,491 (53%) | 1,578 (60%) | 551 (51%) | 1,346 (46%) | 620 (52%) | 396 (56%) | <0.001 |
| **Type of place of residence** |  |  |  |  |  |  | <0.001 |
| Urban | 6,790 (80%) | 2,143 (82%) | 811 (74%) | 2,627 (91%) | 708 (59%) | 501 (71%) |  |
| Rural | 1,727 (20%) | 481 (18%) | 279 (26%) | 271 (9.4%) | 495 (41%) | 200 (29%) |  |
| **Religion** |  |  |  |  |  |  | 0.011 |
| Muslim | 8,483 (100%) | 2,610 (99%) | 1,086 (100%) | 2,897 (100%) | 1,194 (99%) | 696 (99%) |  |
| Non-Muslim | 34 (0.4%) | 15 (0.6%) | 3 (0.3%) | 2 (<0.1%) | 9 (0.7%) | 6 (0.8%) |  |
| **Eligibility for government free services** | 7,499 (89%) | 2,342 (89%) | 1,062 (98%) | 2,339 (84%) | 1,102 (92%) | 654 (93%) | <0.001 |
| **Health insurance coverage** | 1,495 (18%) | 586 (22%) | 141 (13%) | 564 (20%) | 108 (9.0%) | 96 (14%) | <0.001 |
| **Private insurance coverage** | 273 (3.2%) | 82 (3.1%) | 15 (1.3%) | 134 (4.7%) | 23 (1.9%) | 19 (2.7%) | <0.001 |
| **Subjective health care need** | 1,613 (20%) | 594 (26%) | 160 (15%) | 543 (20%) | 198 (17%) | 118 (18%) | 0.001 |
| ^1^n (%) | | | | | | | |
| ^2^chi-squared test with Rao & Scott's second-order correction | | | | | | | |

**Supplementary Table 2: Sociodemographic characteristics of survey respondents with reported NCD diagnosis**

| Characteristic | Overall,  N = 1,453^1^ | Central,  N = 487^1^ | Eastern,  N = 189^1^ | Western,  N = 493^1^ | Southern, N = 211^1^ | Northern, N = 73^1^ | p-value^2^ |
| --- | --- | --- | --- | --- | --- | --- | --- |
| **sex** |  |  |  |  |  |  | 0.6 |
| Male | 785 (54%) | 279 (57%) | 99 (52%) | 257 (52%) | 113 (54%) | 37 (51%) |  |
| Female | 668 (46%) | 209 (43%) | 90 (48%) | 236 (48%) | 98 (46%) | 36 (49%) |  |
| **Age categories** |  |  |  |  |  |  | <0.001 |
| 18-24 years | 55 (3.8%) | 31 (6.3%) | 4 (2.0%) | 13 (2.6%) | 6 (3.0%) | 1 (2.0%) |  |
| 25-34 years | 209 (14%) | 92 (19%) | 10 (5.2%) | 70 (14%) | 28 (13%) | 10 (14%) |  |
| 35-44 years | 341 (23%) | 141 (29%) | 53 (28%) | 86 (18%) | 42 (20%) | 18 (25%) |  |
| 45-54 years | 314 (22%) | 100 (20%) | 44 (23%) | 110 (22%) | 45 (21%) | 15 (21%) |  |
| 55-64 years | 294 (20%) | 69 (14%) | 43 (23%) | 126 (25%) | 46 (22%) | 11 (14%) |  |
| +65 years | 241 (17%) | 56 (11%) | 36 (19%) | 89 (18%) | 44 (21%) | 17 (24%) |  |
| **Marital status** |  |  |  |  |  |  | 0.1 |
| Never married | 105 (7.2%) | 51 (10%) | 11 (5.9%) | 31 (6.3%) | 10 (4.9%) | 2 (3.2%) |  |
| Currently married | 1,058 (73%) | 330 (68%) | 149 (79%) | 357 (72%) | 165 (79%) | 56 (77%) |  |
| Separated/ Divorced | 106 (7.3%) | 48 (9.8%) | 6 (3.2%) | 40 (8.2%) | 9 (4.4%) | 3 (4.0%) |  |
| Widowed | 183 (13%) | 59 (12%) | 23 (12%) | 65 (13%) | 26 (12%) | 11 (16%) |  |
| **Wealth index** |  |  |  |  |  |  | <0.001 |
| 1 | 246 (17%) | 31 (6.4%) | 38 (20%) | 105 (21%) | 58 (27%) | 14 (19%) |  |
| 2 | 307 (21%) | 48 (9.9%) | 44 (23%) | 142 (29%) | 61 (29%) | 12 (16%) |  |
| 3 | 229 (16%) | 52 (11%) | 32 (17%) | 90 (18%) | 42 (20%) | 13 (18%) |  |
| 4 | 291 (20%) | 93 (19%) | 44 (23%) | 104 (21%) | 28 (13%) | 22 (30%) |  |
| 5 | 381 (26%) | 263 (54%) | 30 (16%) | 53 (11%) | 23 (11%) | 12 (16%) |  |
| **Income categories*** |  |  |  |  |  |  | <0.001 |
| Low income | 426 (30%) | 54 (11%) | 75 (42%) | 194 (40%) | 76 (37%) | 26 (36%) |  |
| Lower-middle income | 201 (14%) | 48 (10%) | 44 (25%) | 61 (13%) | 35 (17%) | 14 (20%) |  |
| Middle income | 131 9.3%) | 38 (8.0%) | 19 (11%) | 44 (9.1%) | 22 (10%) | 8 (12%) |  |
| Upper-middle income | 223 (16%) | 83 (18%) | 18 (10%) | 74 (15%) | 37 (18%) | 11 (15%) |  |
| High income | 434 (31%) | 251 (53%) | 21 (12%) | 111 (23%) | 39 (19%) | 12 (17%) |  |
| **Nationality** |  |  |  |  |  |  | 0.04 |
| Saudi | 1,307(90%) | 438 (90%) | 182 (96%) | 428 (87%) | 192 (91%) | 66 (90%) |  |
| Non-Saudi | 147 (10%) | 49 (10%) | 7 (3.7%) | 65 (13%) | 18 (8.7%) | 7 (9.5%) |  |
| **Education level** |  |  |  |  |  |  | <0.001 |
| No formal education | 178 (12%) | 21 (4.4%) | 29 (15%) | 61 (12%) | 55 (26%) | 13 (17%) |  |
| Less than secondary | 313 (22%) | 58 (12%) | 70 (37%) | 107 (22%) | 52 (25%) | 26 (37%) |  |
| Secondary | 325 (22%) | 121 (25%) | 49 (26%) | 98 (20%) | 41 (20%) | 14 (20%) |  |
| More than secondary | 635 (44%) | 285 (59%) | 41 (22%) | 227 (46%) | 63 (30%) | 19 (26%) |  |
| **Employment** |  |  |  |  |  |  | <0.001 |
| Yes | 678 (47%) | 288 (59%) | 80 (42%) | 193 (39%) | 83 (40%) | 33 (46%) |  |
| No | 400 (28%) | 132 (27%) | 36 (19%) | 144 (29%) | 77 (36%) | 12 (16%) |  |
| Never worked before | 375 (26%) | 67 (14%) | 74 (39%) | 156 (32%) | 50 (24%) | 28 (39%) |  |
| **Type of place of residence** |  |  |  |  |  |  | <0.001 |
| Urban | 1,194 (82%) | 415 (85%) | 166 (88%) | 427 (87%) | 128 (61%) | 57 (79%) |  |
| Rural | 259 (18%) | 72 (15%) | 23 (12%) | 66 (13%) | 83 (39%) | 15 (21%) |  |
| **Religion** |  |  |  |  |  |  | 0.14 |
| Muslim | 1,450 (100%) | 487 (100%) | 188 (99%) | 493 (100%) | 209 (99%) | 72 (99%) |  |
| Non-Muslim | 4 (0.3%) | 1 (0.1%) | 1 (0.5%) | 0 (0%) | 1 (0.6%) | 1 (1.1%) |  |
| **Eligibility for government free services*** | 1,314 (92%) | 448 (92%) | 186 (98%) | 416 (89%) | 195 (93%) | 68 (94%) | 0.011 |
| **Health insurance coverage** | 297 (20%) | 163 (33%) | 18 (9.6%) | 89 (18%) | 20 (9.4%) | 7 (9.3%) | <0.001 |
| **Private insurance coverage** | 54 (3.7%) | 25 (5.2%) | 3 (1.5%) | 19 (3.8%) | 4 (2.0%) | 3 (3.9%) | 0.2 |
| **Subjective health care need** | 495 (35%) | 231 (48%) | 41 (22%) | 141 (29%) | 64 (31%) | 18 (25%) | <0.001 |
| ^1^n (%) | | | | | | | |
| ^2^chi-squared test with Rao & Scott's second-order correction | | | | | | | |
